# Work-Related Human T-lymphotropic Virus 1 and 2 (HTLV-1/2) Infection: A Systematic Review

**DOI:** 10.3390/v13091753

**Published:** 2021-09-02

**Authors:** Angela Stufano, Hamid Reza Jahantigh, Francesco Cagnazzo, Francesca Centrone, Daniela Loconsole, Maria Chironna, Piero Lovreglio

**Affiliations:** 1 Interdisciplinary Department of Medicine-Section of Occupational Medicine, University of Bari, 70124 Bari, Italy; hamidreza.jahantigh@uniba.it (H.R.J.); francesco.cagnazzo@uniba.it (F.C.); piero.lovreglio@uniba.it (P.L.); 2 Department of Biomedical Sciences and Human Oncology-Hygiene Section, University of Bari, 70124 Bari, Italy; francesca.centrone@uniba.it (F.C.); daniela.loconsole@uniba.it (D.L.); maria.chironna@uniba.it (M.C.)

**Keywords:** occupational risk, HTLV infection, health care workers, sex workers, blood borne pathogens, STLV-1 zoonotic transmission

## Abstract

Human T-lymphotropic virus 1 and 2 (HTLV-1/2) belong to the delta group of retroviruses which may cause a life-long infection in humans, HTLV-1 leading to adult T-cell leukemia/lymphoma and other diseases. Different transmission modes have been described, such as breastfeeding, and, as for other blood-borne pathogens, unsafe sexual activity, intravenous drug usage, and blood transfusion and transplantation. The present systematic review was conducted to identify all peer-reviewed studies concerning the work-related infection by HTLV-1/2. A literature search was conducted from January to May 2021, according to the PRISMA methodology, selecting 29 studies: seven related to health care workers (HCWs), five to non-HCWs, and 17 to sex workers (SWs). The findings showed no clear evidence as to the possibility of HTLV-1/2 occupational transmission in HCWs, according to the limited number and quality of the papers. Moreover, non-HCWs showed a higher prevalence in jobs consistent with a lower socioeconomic status or that could represent a familial cluster, and an increased risk of zoonotic transmission from STLV-1-infected non-human primates has been observed in African hunters. Finally, a general increase of HTLV-1 infection was observed in SWs, whereas only one paper described an increased prevalence for HTLV-2, supporting the urgent need for prevention and control measures, including screening, diagnosis, and treatment of HTLV-1/2, to be offered routinely as part of a comprehensive approach to decrease the impact of sexually transmitted diseases in SWs.

## 1. Introduction

Human T-lymphotropic virus 1 and 2 (HTLV-1 and HTLV-2) belong to the delta group of retroviruses, together with virus 3 and 4 (HTLV-3, HTLV-4), their simian counterparts (Simian T-lymphotropic viruses, STLVs), and the bovine leukemia virus (BLV) [1].

It has been estimated that 5–10 million people are currently infected by HTLV-1, mainly in the endemic regions, including South Japan, Northeastern Iran, sub-Saharan Africa, almost all of the Caribbean islands, southeastern U.S. regions, Melanesia, and South America [2,3]. HTLV-2 is most common in the native Amerindian population, particularly in Amazon region, with the number of known infected people significantly lower than with HTLV-1, being estimated at between 670,000 and 890,000 people [2,4]. Initially confined to specific geographic areas, HTLV-1 and HTLV-2 are now becoming a major concern also in non-endemic countries, due to international migration flows. HTLV-3 and HTLV-4 have been discovered in central Africa in the past decade and both shared similarities in replication and genomic organization to HTLV-1/2 [5].

Most HTLV-1-infected subjects are asymptomatic, but 2–5% suffer the onset of adult T-cell leukemia/lymphoma (ATL), while a smaller proportion of infected people develops a neurological disorder called HTLV-1-related myelopathy/tropical spastic paraparesis (HAM), or else other chronic illnesses, such as uveitis and dermatitis [6,7]. Unlike HTLV-1, the relationship between HTLV-2 and human diseases is unclear, as only sporadic reports have been made of neurological conditions, such as uncommon progressive myelopathies, and of inflammatory disorders, and leukemia, possibly associated to the infection [8,9].

HTLV-1 exhibits little genetic heterogeneity, being spread by the clonal division of infected cells, rather than by reverse transcription-mediated viral replication. Seven different genomic species (A-G) have been identified, mostly related to the geographic origin; subtypes A, B, and C are known to cause ATL, HAM, and dermatitis [10,11,12]. Serologic testing is most often used for laboratory diagnosis of HTLV-1 infection. Various serologic screening assays are commercially available to detect HTLV-1 and HTLV-2 antibodies present in plasma and sera from donors of blood, human cells, tissues and cellular and tissue-based products, and from patients presenting with signs and symptoms suggestive of HAM, such as a positive enzyme-linked immunosorbent assay (ELISA) andchemiluminescent immunoassay (CLIA) or electro-chemiluminescent immunoassay (ECLIA). Due to the potential for false-positive results in these initial tests, supplementary serologic assays like Western blot or line immunoassay (LIA) are used to confirm the initial results and to differentiate between HTLV-1 and HTLV-2 antibodies. Molecular assays, such as real-time PCR or quantitative PCR (qPCR) have been used to fully determine the status of HTLV infections and quantify the proviral load [13,14].

Transmission of HTLV-1 is lymphocyte-mediated and requires cell-to-cell contact [15]. Several different modes of inter-host HTLV-1 transmission have been described, such as sexual transmission, parenteral transmission via transfusion of contaminated blood products, transplantation of infected organs, or drug injections, contact with blood during scarification in rituals, but vertical transmission is thought to be the main mode maintaining the endemicity, as a result of the passage of infected maternal cells in breast milk of carrier mothers to infants during prolonged breastfeeding [3,16,17]. Moreover, zoonotic transmission of simian T-cell leukemia virus type 1 (STLV-1) to humans after contact with non-human primates (NHPs) through bites or bushmeat slaughtering still occurs in Africa [18]. According to the sexual route of contagion, occupational HTLV-1/2 transmission has been considered probable in sex workers (SWs), whereas a possibly higher risk of occupational infection by biological accident has been postulated for health care workers (HCWs), more because of the similarity to other blood-borne pathogens than based on scientific evidence [3,19]. The present systematic review, therefore, is focused on the available pertinent scientific information concerning the possibility of work-related infection by HTLV-1/2.

## 2. Materials and Methods

**Figure 1:**
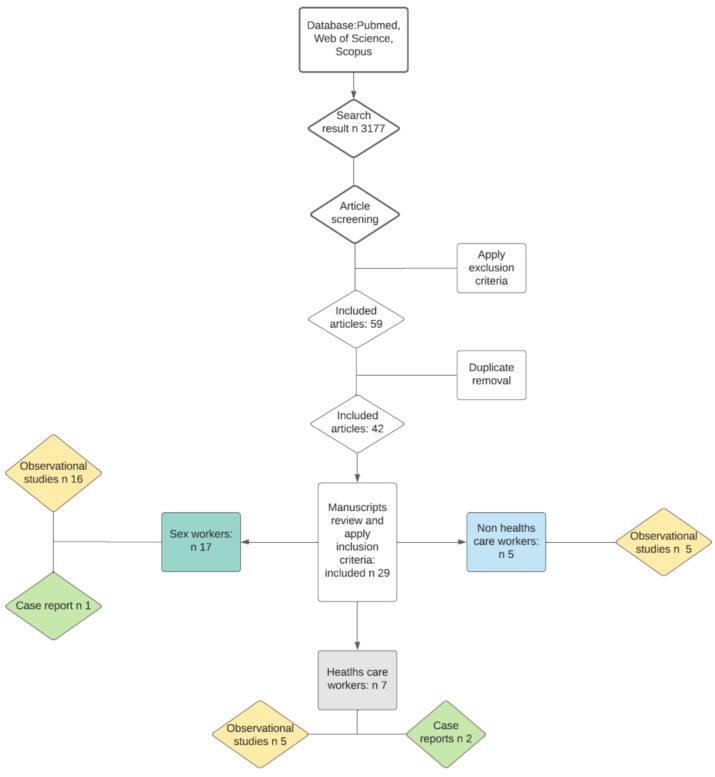
Description of the selection process for articles included in the review (PRISMA method).

A systematic literature search was conducted to select available information according to the PRISMA (Preferred Reporting Items for Systematic Reviews and Meta-Analyses) methodology, to identify all peer-reviewed studies of the relationship between HTLV-1/2 infection and the occupational setting. Firstly, a systematic review of the literature was made using the PubMed, Scopus, Web of Science databases. A search strategy was developed and adapted for each database, and then one researcher performed data extraction from January to May 2021. The variables to be analyzed were selected according to the population, exposure, comparison, and outcome (PECO) method, which is often used for systematic review and is easily adapted to a particular case and combined with other essential variables. Characteristics of individuals (population), exposure to the phenomenon (direction), and their consequences (outcome) were considered. The following keywords: (“HTLV-1 OR HTLV-2) AND (“worker” OR “job” OR “occupational” OR “professional”) were used for the search. Screening of resources took place in the first phase, by reading the articles titles and abstracts and removing duplicates (Figure 1). This phase yielded *n* = 42 selected items.

In the second stage, the full texts of the selected articles were screened independently by two co-authors, based on the inclusion criteria, namely, the English language, case reports or research article, topic on HTLV-1/2 infection or anti-HTLV-1/2 seroprevalence and occupational setting, and exclusion criteria, that were non-occupational setting studies or topics on other viruses. To establish a final set of papers, the two reviewers compared their findings to identify consistencies and discrepancies. Any differences were resolved by mutual agreement. At the end of this second phase, the final selection of *n* = 29 items was made.

The selection for papers on SWs was made according to the World Health Organization (WHO) definition, that considers women, men, and transgendered SWs as those people who receive money or goods in exchange for sexual services, and who consciously define those activities as income-generating, even if they do not consider sex work as their occupation [20].

## 3. Results

The details of the articles included in the review are reported according to the occupational setting, distinguishing HCWs, non-HCW and SWs. 

### 3.1. Health Care Workers

Seven articles on HTLV-1/2 infection in HCWs were collected, including five observational studies and two case-report studies (Table 1).

The study performed by Hewagama et al. [21] retrospectively analyzed, over a 10-year period (2002–2012), seroconversion for HTLV-1/2 in 53 Central Australian HCWs monitored by serologic follow-up after biological accidents while treating HTLV-1/2 positive patients. The information was drawn from the regional infection control database and the Australian Northern Territory Government Pathology Service. No evidence of seroconversion was observed in any of the HCWs, although post-exposure prophylaxis was performed only in three cases. One possible limitation reported by the Authors of the study, however, was that although time of HTLV-1/2 seroconversion is not known, it could occur between 2 and 3 months after the infection, according to the data observed after transfusion with infected blood, whereas the follow-up period was not longer than 120 days in more than half of the workers (median 117 days) [17].

In a survey performed from 2000 to 2008 to investigate the epidemiology of HTLV-1/2 in US Armed Force military personnel in order to ensure emergency transfusion safety, a higher rate of HTLV-1/2 diagnosis was found in military HCWs (0.94 per 100,000 person-years) vs. combat military personnel (0.37 per 100,000 person-years) and other military personnel (0.54 per 100,000 person-years), yielding a relative risk (RR) for HCWs vs. combat personnel of 2.54 (95% CI 1.1, 6.1) [22].

Stuver et al. [23] observed an 8.5% HTLV-1 seroprevalence overall in 7055 individuals from the Miyazaki endemic region (Japan), in the period September 1983–December 1984. Analysis of the prevalence by job showed a significantly higher RR for HTLV-1 infection (corrected for age, sex and area) in fishermen (RR = 3.0; 95% CI 1.1, 8.3), forestry workers (RR = 2.5; 95% CI 1.2, 5.2), and workers raising livestock (RR = 2.0; 95% CI 1.3, 3.1), while no increased risk of infection was observed for HCWs (RR = 0.92; 95% CI 0.5, 1.7). In view of the study design, the higher prevalence of HTLV-1 for some jobs seemed to be related more to the influence of intrafamilial transmission than to occupational exposure.

A seroprevalence survey was conducted by Goubau et al. [24] among 42 HCWs and 158 patients at the hospital of Lisala (Congo Democratic Republic), located in a high HTLV-1/2 prevalence area, where the first African focus of HAM was described, showing a typical familial and ethnic clustering of the cases. Six of the 42 HCWs investigated (14.3%) were positive to HTLV-1/2, showing a prevalence not significantly different from that in patients (13.9%).

A HTLV-1 seroprevalence study, including 291 intravenous drug abusers, 45 household contacts of surveyed drug abusers, and 39 laboratory HCWs at a methadone maintenance center in Rome (Italy), was conducted between 1985 and 1987 [25]. Serological screening showed the presence of HTLV-1 antibodies in 6.6% of the drug abusers, but in none of the household contacts and laboratory personnel, indicating the lack of an occupational risk for these HCWs.

Barreto [26] reported the case of a 29-year-old Caucasian female laboratory HCW who had sustained a biological accident while recapping a needle. The source patient was a 32-year-old African-Brazilian male, intravenous drug user, infected by HTLV-2 and probably by HCV. The worker’s serological tests for blood-borne viruses, including HTLV-1/2, were all normal at baseline, but 18 months after the accident, HTLV-1/2 serologic tests yielded a positive result, confirmed for HTLV-2 by WB assay and PCR test. No other risk factors for HTLV-1/2 infection were identified for the worker and all the relatives tested negative for HTLV-1/2, except for a 6-month-old daughter, still being breastfed, who showed a weak-positive ELISA test result, probably due to the transmission of maternal antibodies, but negative PCR.

A case of HTLV-1 associated to a HAM was described by Goubau et al. [27] in a 57-year-old Belgian nun who had been working as a midwife in Zaire (actually RD Congo). Occupational exposure was the only risk factor identified. She referred that she did not wear gloves while caring for patients during delivery, thus undergoing abundant exposure to blood over many years.

### 3.2. Non-Health Care Workers

Five observational studies on work-related HTLV-1/2 prevalence in non-healthcare occupational settings were collected and included in the review (Table 2).

A cross-sectional survey made in 2005–2012 compared the HTLV-1 prevalence in 269 individuals (254 men, 15 women) from rural villages and settlements in Cameroon (exposed), bitten by NHPs mostly during hunting activities, vs. 269 matched subjects from the same settlers, but referring no previous NHPs attacks (controls) [28]. The study aim was to investigate the possible interspecies transmission from NHPs to humans of STLV-1, the virus from which HTLV-1 could have originated. The HTLV-1 prevalence was 8.6% (23/269) in the exposed group, associated to the bite severity, vs. 1.5% (4/269) in controls (*p* < 0.001). All the HTLV-1-positive hunters bitten by a gorilla or chimpanzee were infected by a subtype B strain, like that in monkeys in the same area, whereas two hunters attacked by small monkeys were infected by subtype F, that was very close to the strains found in those small monkeys. Mother-to-child infection was excluded in the six infected subjects in whom transmission could be investigated, clearly indicating that hunting NHPs should be considered an occupational activity at risk of zoonotic transmission by STLV-1.

A similar cross-sectional study was performed by Kazanji et al. [29] in southeastern Gabon, investigating 78 people (10 women, 59 men, and 9 children) reporting a history of severe bites and scratches from NHPs while hunting or playing with animals, and 85 individuals from the same village, who had not suffered NHPs bites (controls). Among the 78 participants, seven people (9.0%) were positive for HTLV-1 vs. 22 (25.9%) among the controls. Notably, one child severely bitten by a *Cercopithecus nictitans* resulted in infection by a subtype D strain that was closely related to the STLV-1 that infected this monkey species, while her mother was infected by a subtype B strain. The observed results, therefore, confirmed that hunters in Africa should be considered a high-risk population, because a severe NHPs bite is a risk factor for STLV-1 acquisition.

Few other studies have investigated work-related HTLV-1/2 infection in non-HCWs. No cases of HTLV-1 infection were assessed in 28 Mexican slaughterhouse workers in a survey performed to rule out cross-reactivity with BLV [30]. Norrgren et al. [31] assessed the seroprevalence of HTLV-1/2 in 1377 police officers (1234 males and 143 females) from Guinea Bissau in the period January 1990–December 1992. The overall prevalence of infection was 4.4% (4.0% for HTLV-1 and 0.4% for HTLV-2). Moreover, the HTLV-1/2 incidence during the follow-up of 515 negative workers (mean follow-up time 19.2 months) showed only two new HTLV-1 cases and one new HTLV-2 infection. A further observational study was conducted in the period 1985–1986, to analyze the HTLV-1 prevalence in a cohort of 13,260 Jamaican people who applied for food handling licenses, including both current employees and those seeking employment [32]. The overall HTLV-1 prevalence was 6.1%, being highest for skilled tradesmen in males (4.7%), and for farmers and laborers (10.5%) and self-employed workers (9.8%) in females. Logistic regression, with the professional/student occupation as the reference category, showed that all the other jobs (domestic, tradesman, self-employed, unemployed, farmer/laborer) had significant ORs, ranging from 1.92 to 2.48. Moreover, an OR lower for professionals/students than for the other jobs (OR 0.42) was also observed including in the model age, sex and ethnicity. Both the studies by Norrgren et al. and Murphy et al. performed a screening test campaign followed by a confirmatory procedure by WB.

### 3.3. Sex Workers

Seventeen articles related to the SWs were collected, including 16 observational studies and one case report (Table 3).

A cross-sectional study was conducted from 2005 to 2006 in four cities in the State of Parà (Brazil) amongst 339 female SWs, to analyze sociodemographic characteristics, sexual habits, frequency, circulating molecular subtypes of HIV-1 and the HTLV-1/2 seroprevalence [33]. The HTLV-1 prevalence was 1.8%, while no HTLV-2 infection was detected. Unprotected sex (OR 9.5; 95% CI 1.1–61.1) and illicit drug use (OR 7.1; 95% CI 1.4–34.2) were associated with HTLV-1 infection.

A recent study by Paulino-Ramirez et al. [34] in the period December 2012–April 2013 investigated the HTLV-1/2 seroprevalence in a population of 200 Santo Domingo (Dominican Republic) individuals, including 79 transactional SWs (29 males, 50 females) and 119 intravenous drug users (70 males and 49 females); some of them reported both conditions. The HTLV-1/2 prevalence was 27.6% in male (8 cases) and 10% in female (5 cases) SWs, higher than that observed in intravenous drug users (14.3% in males and 4.1% in females). However, HTLV infection was not associated with sex work (OR 1.79; 95% CI 0.77–4.15).

A cross-sectional study investigated HTLV-1/2 co-infection, between April 2015 and December 2017, in the 21 female SWs showing HBV infection among a total group of 153 female SWs from the Marajo Archipelago (Northern Brazil) [35]. No HTLV-1 positivity was detected, while one case of HBV–HTLV-2 co-infection was identified (4.8%).

Data collected in 1938 female SWs from Callao (Perù), screened for HTLV-1 infection over three study periods between 1993 and 2010, were used to examine the trend in HTLV-1 prevalence [36]. The overall HTLV-1 prevalence was 9.6%, and decreased significantly from 1993 (14.5%) to 2010 (3.1%) (*p* < 0.01), no cases of HTLV-1 being detected among the 224 SWs born after 1979. Among the investigated variables, age, earlier birth cohort, native home in the Andes, duration of sex work, age at the time of starting sex work, as well as HIV seropositivity, were positively associated with HTLV-1 infection.

The report by Bautista et al. [37] investigated the prevalence of HTLV-1/2 in 625 female SWs from Argentina, during the period 2000–2002, including immigrants (27.0%) and non-immigrants (73.0%). No significant difference in HTLV-1/2 prevalence was observed between the non-immigrant and immigrant SWs groups either for HTLV-1 (1.3% vs. 1.8%) or HTLV-2 (0.2% vs. 0.0%).

The prevalence of HTLV-1/2 infection amongst 166 female SWs, 120 pregnant women (PW) and 78 female secondary school students from Ibadan (South-Western Nigeria) was assessed by Forbi et al. [38]. The overall prevalence of HTLV antibodies was significantly higher in SWs (22.9%) than in PW (16.7%) and students (5.1%).

Berini et al. [39] studied the HTLV-1/2 prevalence during the period 2000–2003 in 2055 high-risk subjects from Argentina, including 613 female SWs, 173 injecting drug users (IDUs), 682 men who had sex with men (MSM), 187 tuberculosis patients (TB) and 400 patients attending clinics for sexually transmitted diseases (STD). The HTLV-1/2 prevalence was 2.0% for SWs (1.5% HTLV-1, 0.2% HTLV-2), 19.1% for IDUs (4.6% HTLV-1, 15.6% HTLV-2), 2.1% for TB (1.6% HTLV-1, 0.5% HTLV-2), 1.0% for STD and 0.4% for MSM (all HTLV-1).

Another cross-sectional study was conducted between March 2000 and March 2002 by Pando et al. [40] among 614 female SWs from six different cities in Argentina, to estimate the seroprevalence of HTLV-1/2 that resulted 1.6% as overall value, HTLV-1 positivity being observed in 7 of 10 HTLV-1/2 positive samples. A higher prevalence of HTLV-1 infection was found among Argentinian SWs than among those from other countries (OR 28.3; 95% CI 4.9 to 154.1) and HTLV-1/2 and HBV infection were found to be significantly associated (OR 12.0; 95% CI 1.9 to 96.1).

A survey conducted by Zehender et al. [41] between March 1996 and September 2003 analyzed the prevalence of HTLV-1/2 infections in 167 HIV positive immigrants in Milan (Italy), including 52 male-to-female transsexual SWs, and 226 pregnant HIV-1 negative women, enrolled as the control group. The study aim was to ascertain whether the infections observed were due to a recent spread among high-risk subjects or had been transmitted independently in the countries of origin. All six HTLV-1–HIV co-infected individuals were among the transsexual SWs, showing an HTLV-1 prevalence of 11.5%, while in the HIV-negative control group, an HTLV-1 prevalence of 0.9% was observed. Particularly, all the HTLV-1 cases were found in subjects from Latin America, mostly born in Peru (26.3% in the HIV-1 positive group). HTLV-2 was only found in two HIV-1 positive transsexual SWs from Brazil, showing an overall prevalence of 6.4%. Phylogenetic analysis suggested the independent origin of each infection in the patient’s birthplace.

Trujillo et al. [42] reported a survey conducted in Lima (Perù) from March to June 1994, to determine the relationship between sexual behavior practices and the prevalence of HTLV-1/2 infections among 158 unlicensed female SWs. The HTLV-1 seroprevalence was 3.7%, while all subjects were negative for HTLV-2. The prevalence of HTLV-1 infection was significantly lower among SWs who always used condoms (OR 0.15; 95% CI, 0.03–0.085).

Between 1993 and 1996, Chen et al. [43] studied the prevalence of HTLV-1/2 in female SWs from Taipei (Taiwan), observing a total HTLV-1 prevalence of 0.61% (2/328) for SWs at a massage parlor, 1.30% (10/770) at a karaoke bar and 4.23% (12/284) at a brothel. No HTLV-2 seropositive individuals were found. No significant change was found over time in the HTLV prevalence rate among the three SWs groups tested.

An epidemiologic study was conducted by Zurita et al. [44] to determine the frequency of HTLV-1/2 infection among adults of Quechua origin in Cuzco and Quillabamba (Peru), namely 51 female SWs, 211 healthy PW, 47 suspected STD patients, 48 homosexual/bisexual individuals and 13 promiscuous heterosexual males. The HTLV-l seropositivity rate was 13.7% for female SWs, significantly associated with a history of STD, higher than for PW (2.3%), STD patients (8.5%), homosexual/bisexual individuals (6.2%), promiscuous heterosexual males (0.0%). No cases of HTLV-2 infection were observed.

A cross-sectional survey of the HTLV-1/2 seroprevalence was performed in North-East Brazil, in the period July 1993–February 1994 in 496 female and 171 male SWs, 814 PW, 494 TB and 395 STD patients, 427 prisoners [45]. The prevalence of HTLV-1 was 1.21% in female and 0.58% in male SWs, higher than in PW (0.12%), TB patients (0.44%), STD patients (0.50%) and prisoners (0.47%). Only one female SW resulted positive for HTLV-2, the same as in PW and TB patients, while in prisoners there were two positive HTLV-2 cases, but among male SWs and STD patients no cases were found.

A cross-sectional observational study by Bellei et al. [46] evaluated the HTLV-1/2 seroprevalence in 653 female SWs and 153 male sexual clients living in the city of Santos (Brazil). The HTLV-1 seropositivity rate was 2.8% in the SWs and 2.0% in their clients. Infection by HTLV-2 could not be demonstrated with a WB confirmatory test. Amongst the SWs, the frequency of anti-HTLV-1 antibodies was three-fold that in those with a history of blood transfusion in the previous five years.

In a cross-section observational study, Zapata-Benavides et al. [47] performed HTLV-1/2 screening of serum samples collected in six Mexican states from 75 female SWs, 335 patients with leukemia or lymphoma, 103 other cancer patients, 387 with multiple blood transfusions, 87 homosexuals, 90 HIV positive patients, and one with multiple sclerosis. None of the 1087 individuals from the groups at risk demonstrated confirmed HTLV-1/2 positivity.

Yoshida et al. [48] investigated the HTLV-1 prevalence in 237 female SWs in Fukuoka city (Japan), during a three-month study period in 1986. The HTLV-1 prevalence in the SWs was 5.9%, within the usual range for the Kyushu district where the study was performed.

A case-report of HTLV-1 infection was described by Caterino-De-Araujo et al. [49] in a 25 year-old female SW from Imbituba (Santa Catarina, Brazil), with no HTLV-1-associated disease, no steady partner, one month working as a prostitute with one client per day, vaginal and oral sexual practices, no use of condoms, no blood transfusion. The case-report suggested that sex work must have been the major route of virus acquisition.

A comparison between the prevalence of HTLV-1/2, HIV-1 and other STDs in SWs, has been described in 11 of the 16 observational studies included in the review (Table 4).

## 4. Discussion

The present review investigated the relationship between different working activities and infection by HTLV-1/2 to probe whether prevention in workplaces should play a role in the global approach to the prevention of the diseases caused by HTLV-1/2.

Previous studies have often considered HCWs at higher risk of HTLV-1/2 infection, according to the possible virus transmission by the parenteral route following biological accident [50]. Our systematic review, however, showed that there is no clear scientific evidence as to the possibility of HTLV-1/2 occupational infection in HCWs. In fact, an occupational origin was suspected in two case reports, with only one patient showing seroconversion, whereas only one of the sero-epidemiological studies included in the review showed an increased HTLV-1/2 prevalence in HCWs [22,26,27]. However, the observational studies on the topic were few and showed strong limitations, such as the size of the study sample, a control group not representative for the general population, or no HTLV confirmation assay as in Stuver et al. [23]. Moreover, only one study investigated HCWs seroconversion after biological accidents with HTLV infected patients according to the Northern Territory (Australia) policy, the only one that introduced a specific procedure for the management of occupational exposure after HTLV-1 biological accidents, including testing for the source patient and follow-up serology on the recipient [3]. This study showed negative results too, although these cannot be considered conclusive due to severe limitations [21].

A possible explanation for the different behavior between HTLV-1/2 and the other blood-borne viruses could be related to the fact that HTLV-1/2 are cell-associated viruses, that probably require the transfer of whole cells for virus transmission to occur. In fact, unlike other retroviruses, lymphocytes infection by free virions is very inefficient, as it is for most cell types except dendritic cells, while transmission is greatly improved upon the establishment of cell-cell contact [51]. A minimal number of 90,000 infected cells was estimated to be required for the infection to occur, and this may explain why a high percentage of individuals was infected after receiving HTLV-1 infected cellular blood components, but not after receiving non-cellular blood products (plasma fraction or plasma derivatives) from infected individuals [52]. It would be possible, therefore, that rarely biological accidents while treating infected patients may allow interaction with a sufficient amount of cellular components [53]. Moreover, HTLV-1/2 transmission is dependent on the pro-viral load in the source of the possible contagion, that is negligible in a large number of HTLV-1/2 infected subjects, even if untreated. Further studies, therefore, seem to be essential to assess the risk of HTLV-1/2 transmission by biological accidents, also considering that the efficacy of post-exposure prophylaxis with HIV antiretroviral therapy in preventing the occupational transmission of HTLV-1 remains unknown [3].

The prevalence of HTLV-1/2 infection in occupational groups other than HCWs was investigated in few cross-sectional studies, most of which were conducted in endemic areas, over a period ranging from 1990 to 2015. Firstly, a higher HTLV-1 prevalence was observed in Central Africa hunters, where interspecies transmission of STLV-1 by NHPs has been considered as a possible source of infection. Molecular characterization of primate T-lymphotropic viruses, both simians and humans, reveals that some HTLV subtypes share closer genetic ties to certain STLVs than to other HTLV subtypes, suggesting sustained zoonotic transmission of STLV between NHPs and humans [54]. Phylogenetic and epidemiologic evidence from Central Africa supports the notion that crossover events of interspecies transmission of STLV to humans have occurred [55]. In these areas, where several NHPs are infected by STLV-1, contact of an uninfected hunter with NHPs body fluids during hunting, collection or the consumption of bush meat, might eventually lead to a zoonotic STLV-1 transmission. This is possible especially in cases of severe bites by NHPs, because the STLV-1 contaminated fluid may include a certain number of infected lymphocytes originating from pre-existing small injuries in the mouth of the animal [56]. HTLV-1, HTLV-3, and HTLV-4 have all been shown to originate from closely related STLVs (STLV-1, STLV-3, and STLV-4, respectively) and phylogenetic analysis shows that all HTLV-1 subtypes except for cosmopolitan subtype A, likely have primate origins, suggesting that direct zoonotic transmission may account for a measurable proportion of HTLV infections other than intra-familial transmission, at least in the rural regions bordering NHPs habitats [57,58].

Among the reviewed studies, a higher rate of HTLV-1 seropositivity was observed in farmers, laborers and skilled tradesman in a Jamaican population, and in fishing, livestock and forestry workers in a high prevalence Japanese area [23,32]. However, the observed associations may not necessarily reflect an occupational risk, but could more probably be related to the influence of social behaviors in the working class. In fact, for the Jamaican occupational groups, an apparent trend toward a higher prevalence in jobs consistent with a lower socioeconomic status was observed, while Japanese fishermen could represent a familial rather than an occupational cluster, also in view of the geographic isolation of these areas. The job as a fisherman is passed down through generations, so its relationship to HTLV-1 infection is probably associated with vertical transmission from mother to child. Finally, HTLV-1 prevalence increases with age that, therefore, could have been a role in the results observed in the Jamaican population.

Our literature review showed a general increase of HTLV-1 infection among SWs, whereas only one paper described an increased prevalence for HTLV-2, with all the studies, except that by Forbi et al., including confirmatory tests in the analytical procedure (Table 3). Overall, the HTLV-1/2 prevalence was very different among the SWs populations investigated, although these differences do not seem to be related either to the geographic area where the surveys were conducted, or to the years in which the studies were performed, ranging from 1986 to 2017. However, a decreasing trend of HTLV-1 prevalence was observed in Peruvian female SWs from 1993 to 2010 and no further cases among SWs born after 1979. The increasing availability of free condoms in the late 1980s is likely the cause of this decline over the last two decades, in a population previously considered to be among the most endemic in the world [36].

The high prevalence of HTLV-1 observed in SWs confirmed the epidemiologic importance of sexual transmission, suggesting the essential role of SWs in maintaining the infection and transmission to the general population. The mechanism of transmission in the context of sexual intercourse explains why transmission is more effective from men to women. HTLV-1/2 transmission, in fact, is related to infected T-cell lymphocytes present in the semen that come in contact with the female genital mucosa, mainly composed of epithelial cells which can capture HTLV-1/2 at the apical face and release them at the basal face through transcytosis. HTLV-1 could then infect dendritic cells underneath the epithelial barrier [59]. Vice versa, in the case of transmission from an infected woman to a man, the presence of facilitating cofactors seems to be necessary, such as the presence of female genital lesions that allow contact between infected blood cells, or co-infections (e.g., from Treponema Pallidum), that increase the concentration of lymphocytes in vaginal secretions [39,45]. Supporting these transmission models, no cases of HTLV-1/2 were observed in Brazilian promiscuous heterosexual males, while the only study performed also in sexual clients reported an HTLV-1/2 prevalence of 2.0%, slightly lower than the 2.8% observed in female SWs.

Analysis of the variables influencing the HTLV-1/2 prevalence showed higher rates of seropositivity among SWs who referred to a history of blood transfusions or illicit drug use, and in those reporting low condom use [33,42]. Moreover, the prevalence of infection seems to be related to increasing age, duration of sex work, age at the time of starting sex work, number of partners per day [33,35,36]. Generally, the prevalence of HTLV-1/2 was lower than that of HIV and other STD, except for Japanese and Taiwan SWs, and in the paper by Trujillo et al. [42], whereas co-infection of HTLV-1/2 with HIV or other STD was also described.

## 5. Conclusions

To the best of our knowledge, this is the first systematic literature review to analyze the risk of work-related infection by HTLV-1/2. Although a higher risk of infection has been supposed for HCWs, our review did not identify a clear increased rate of seropositivity in HCWs than in the general population, also due to the severe limitations of the observational studies, whereas the main evidence of infection was referred to case-reports in which inadequate use of personal protective equipment had been reported. Definite conclusions, therefore, are not possible on the basis of the actual knowledge and further studies, particularly focusing on HCWs with history of exposure to biological fluids of HTLV infected patients, are needed to clarify the real extent of the occupational risk for HCWs. An increased risk of zoonotic transmission has been observed in Central Africa hunters who perform their activities in STLV-1 infected NHPs habitats, but epidemiological evidence in larger populations is still scarce. Finally, our review confirms that SWs have a higher risk of contracting mainly HTLV-1, although the limit of the large variability observed in HTLV-1 prevalence, supporting the urgent need for prevention and control measures, including screening, diagnosis and treatment of HTLV-1/2, to be offered routinely as part of a comprehensive approach to decrease the impact of STDs in SWs [60]. Neglect of the fact that SWs may constitute a reservoir of HTLV-1 infection may be causing the spread of this virus in the general population.

## Figures and Tables

**Table 1:** Studies on work-related HTLV-1/2 infection in health care workers (HCWs).

Study	Type of Study (Year)	Country	Occupational Study Population	Analytical/Diagnostic Method	Outcome
Hewagama et al. (2014) [21]	Observational retrospective study (2002–2012)	Central Australia	53 HCWs monitored after biological accident with HTLV-1/2 infected patient	Serodia particle agglutination assay (2002–2008) and CLIA (2009–2012), confirmed by WB	No HTLV-1/2 seroconversion
Petruccelli et al. (2014) [22]	Observational study (2000–2008)	US	Military HCWs vs. combat vs. other military personnel	Diagnostic code for HTLV-1/2, recorded at medical encounter	-HTLV-1/2 rate: HCWs 0.94 vs. combat 0.37 vs. other 0.54 per 100,000 p-yrs. -RR HCWs vs. combat: 2.54
Stuver et al. (1992) [23]	Observational study (1983–1984)	Japan	7055 individuals screened at an Health Promotion Center	IFA	Higher HTLV-1 prevalence for fishing (RR 3.0), forestry (RR 2.5) and livestock (RR 2.0) workers, but not for HCWs (RR 0.92)
Goubau et al. (1990) [24]	Observational study	Congo DR	42 hospital HCWs vs. 158 patients	ELISA confirmed by IFA and WB	HTLV-1/2 prevalence: no difference between HCWs (14.3%) and patients (13.9%)
Titti et al. (1988) [25]	Observational study (1985–1987)	Italy	39 laboratory HCWs at a methadone maintenance center	ELISA confirmed by WB	No HTLV-1 seropositivity in the laboratory HCWs
Barreto (2006) [26]	Case report (1995)	Brazil	29 year-old Caucasian female laboratory HCW	ELISA confirmed by WB	HTLV-2 seroconversion 18 months after biological accident with HTLV-2 infected patient
Goubau et al. (1992) [27]	Case report (1989)	Congo DR	57 year-old Belgian Caucasian female nurse/midwife	IFA confirmed by WB	Possible occupational HTLV-1 infection (no other risk factors)

WB: Western Blot assay; IFA: immunofluorescent assay; p-yrs: person-years; RR: relative risk.

**Table 2:** Studies on work-related HTLV-1/2 infection in non-health care workers.

Study	Type of Study (Year)	Country	Occupational Study Population	Analytical/Diagnostic Method	Outcome
Filippone et al. (2015) [28]	Observational study (2005–2012)	Cameroon	269 hunters (254 men, 15 women) bitten by NHPs vs. matched controls with no NHPs bites reported	WB confirmed by PCR	HTLV-1 prevalence: 8.6% in hunters (linked to bite severity) vs. 1.5% in controls
Kazanji et al. (2015) [29]	Observational study	Gabon	78 individuals (mainly hunters) severely bitten by NHPs vs. 85 individuals from the same village with no NHPs bites reported.	ELISA confirmed by WB and PCR (proviral load measure).	HTLV-1 prevalence: 9.0% in NHPs bitten vs. 25.9% in controls.
Zamora-Avila et al. (2013) [30]	Observational study	Mexico	28 slaughterhouse workers	Passive agglutination assay	No cases of HTLV-1 infection
Norrgren et al. (1995) [31]	Observational study (1990–1992)	Guinea Bissau	1377 police officers (1234 men, 143 women), 515 of them performed follow-up (mean time 19.2 months)	ELISA confirmed by WB	-HTLV-1 prevalence: 4.0%, HTLV-2: 0.4%; -follow up: 2 HTLV-1 and 1 HTLV-2 new cases
Murphy et al. 1990 [32]	Observational study (1985–1986)	Jamaica	13,260 subjects (4372 men, 8888 women) applying for food handling licenses	ELISAconfirmed by WB	HTLV-1 prevalence: ORs higher in domestic (1.92), tradesman (1.97), self-employed (2.06), unemployed (2.26), farmer/laborer (2.48), vs. professional/student occupation.

WB: Western Blot assay; NHPs: non-human primates.

**Table 3:** Studies on work-related HTLV-1/2 infection in sex workers (SWs).

Study	Type of Study	Country	Study Population	Method	Outcome
De Souza et al. (2020) [33]	Observational study (2005–2006)	Brazil	339 female SWs	ELISA confirmed by WB and PCR	-HTLV-1 prevalence: 1.8% -HTLV-2 prevalence: 0.0% -HTLV-1 infection associated with unprotected sex (OR 9.5) and illicit drug use (OR 7.1).
Paulino-Ramirez et al. (2019) [34]	Observational study (2012–2013)	Dominican Republic	79 transactional SWs (29 males, 50 females) and 119 IDU (70 males, 49 females), some reporting both the conditions.	ELISA confirmed by WB	-HTLV-1/2 prevalence: 27.6% in male and 10% in female. -HTLV infection not associated with sex work
Frade et al. (2019) [35]	Observational study (2015–2017)	Brazil	21 HBV positive female SWs	EIA confirmed by PCR	-No HTLV-1 co-infection -one HTLV-2 co-infection.
Stewart et al. (2017) [36]	Observational study (1993–2010)	Perù	1938 female SWs.	EIA confirmed by WB	-HTLV-1 prevalence: 9.6%; -decreasing trend from 1993 (14.5%) to 2010 (3.1%) -no HTLV-1 cases among the 224 SWs born after 1979.
Bautista et al. (2009) [37]	Observational study (2000–2002)	Argentina	625 immigrants (27%) and non-immigrants (73%) female SWs.	ELISA and particle agglutination assay, confirmed by WB	-HTLV-1 prevalence: non-immigrants 1.3% vs. immigrants 1.8%; -HTLV-2 prevalence: non-immigrants 0.2% vs. immigrants 0.0%.
Forbi et al. (2007) [38]	Observational study	Nigeria	166 female SWs vs. 120 PW vs. 78 female secondary school students	micro-ELISA system	HTLV-1/2 prevalence: 22.9% SWs vs. 16.7% PW vs. 5.1% students.
Berini et al. (2007) [39]	Observational study (2000–2003)	Argentina	613 female SWs vs. 173 IDUs, 682 MSM, 187 TB and 400 STS	ELISA and PAA, confirmed by WB and PCR.	HTLV-1/2 prevalence: 2.0% SWs (1.5% HTLV-1, 0.2% HTLV-2), 19.1% IDUs (4.6% HTLV-1, 15.6% HTLV-2), 2.1% TB (1.6% HTLV-1, 0.5% HTLV-2), 1.0% STIs and 0.4% MSM (all HTLV-1).
Pando et al. (2006) [40]	Observational study (2000–2002)	Argentina	614 female SWs	ELISA and PAA confirmed by WB	-HTLV-1/2 prevalence: 1.6% (HTLV-1 7/10 cases); -HTLV-1 prevalence: higher in Argentinian vs. other countries SWs (OR 28.3).
Zehender et al. (2004) [41]	Observational study (1996–2003)	Italy	52 male-to-female transsexual SWs among 167 HIV-1 positive immigrants vs. 226 PW HIV-1 negative immigrants (controls).	ELISA confirmed by WB and PCR	-HTLV-1 prevalence: 11.5% SWs vs. 0.9% controls. -HTLV-2 prevalence: 6.4% SWs vs. 0.0% controls.
Trujillo et al. (1999) [42]	Observational study (1994)	Peru	158 female SWs	ELISA confirmed by WB	-HTLV-1 prevalence 3.7%; -HTLV-2 prevalence 0.0%.
Chen et al. (1998) [43]	Observational study (1993–1996)	Taiwan	328 massage parlor, 770 karaoke bar and 284 brothel female SWs	ELISA confirmed by WB	-HTLV-1 prevalence: 0.61% massage parlor, 1.30% karaoke bar, 4.23% brothel SWs. -No HTLV-2 infection
Zurita et al. (1997) [44]	Observational study	Perù	51 female SWs vs. 211 healthy PW, 47 suspected STD patients, 48 homosexual/bisexual individuals, 13 promiscuous heterosexual males.	ELISA confirmed by WB	-HTLV-l prevalence: 13.7% SWs, vs. 2.3% PW, 8.5% STD patients, 6.2% homosexual/bisexual individuals, 0.0% promiscuous heterosexual males. -No HTLV-2 infection
Broutetet et al. (1996) [45]	Observational study (1993–1994)	Brazil	496 female and 171 male SWs vs. 814 PW, 494 TB and 395 STD patients, 427 prisoners	ELISA confirmed by WB	-HTLV-1 prevalence: 1.21 female and 0.58% male SWs vs. 0.12% PW, 0.44% TB patients, 0.50% STD patients and 0.47% prisoners. -HTLV-2 prevalence: 0.20% female and 0.0% male SWs vs. 0.12% PW, 0.20% TB patients, 0.0% STD patients, 0.47% prisoners.
Bellei et al. (1996) [46]	Observational study (1987–1990)	Brazil	653 female SWs vs. 153 male sexual clients	EIA confirmed by WB	-HTLV-1 prevalence: 2.8% SWs vs. 2.0% their clients. -No HTLV-2 infection.
Zapata-Benavides et al. (1996) [47]	Observational study	Mexico	75 female SWs vs. 335 leukemia/lymphoma patients, 103 other cancer patients, 387 with multiple blood transfusions, 87 homosexuals, 90 HIV positive, 1 with multiple sclerosis.	PAA confirmed with WB and PCR	-No confirmed HTLV-1/2 infection
Yoshida et al. (1987) [48]	Observational study (1986)	Japan	237 female SWs	Serum screening with PAA	-HTLV-1 prevalence: 5.9%
Caterino-De Araujo et al. (2006) [49]	Case Report	Brazil	Female SW	EIA confirmed by WB	Sex work identified as the major via of virus acquisition

EIA: enzyme-linked immunosorbent assay; PAA: particle agglutination assay; IDU: intravenous drug users; PW: pregnant women; MSM: male having sex with male; TB: tuberculosis patients; STS: sexually transmitted diseases patients.

**Table 4:** Comparison among HTLV and other STDs prevalence in SWs studies.

Study	Country	Study Population	HTLV Prevalence	HIV Prevalence	Other STDs Prevalence
De Souza et al. (2020) [33]	Brazil	339 female SWs	-HTLV-1: 1.8% -HTLV-2: 0.0%	-HIV-1: 2.4%	-
Stewart et al. (2017) [36]	Perù	1938 female SWs.	-HTLV-1: 9.6%	-HIV: 0.5%	-
Bautista et al. (2009) [37]	Argentina	625 non-IM (73%) and IM (27%) female SWs.	-HTLV-1: non-IM 1.3% vs. IM 1.8%; -HTLV-2: non-IM 0.2% vs. IM 0.0%.	-HIV-1: non-IM 3.9% vs. IM 1.2%	-HBV: non-IM 12.6% vs. IM 19.4% -HCV: non-IM 5.5% vs. IM 1.2% -*TP*: non-IM 51.5% vs. IM 30.3%
Berini et al. (2007) [39]	Argentina	613 female SWs	-HTLV-1: 1.5%, -HTLV-2: 0.5%	-HIV: 2.9%	-HBV: 14.2% -HCV: 4.2% -*TP*: 43.6%
Pando et al. (2006) [40]	Argentina	614 female SWs	-HTLV-1: 1.1%, -HTLV-2: 0.5%	-HIV: 3.2%	-HBV: 14.4% -HCV: 4.3% -*TP*: 45.7%
Trujillo et al. (1999) [42]	Peru	158 female SWs	-HTLV-1: 3.7%; -HTLV-2: 0.0%.	HIV-1: 0.0%	-*TP*: 3.0%
Chen et al. (1998) [43]	Taiwan	328 massage parlor, 770 karaoke bar and 284 brothel female SWs	-HTLV-1: 0.6% massage parlors, 1.3% karaoke bars, 4.2% brothel. -HTLV-2: 0.0% all settings	HIV-1: 0.2% karaoke bars, 0.0% other settings.	-*TP*: 4.1% massage parlors, 3.4% karaoke bars, 34.9% brothels. -HSV-2: 2.4% massage parlors, 7.5% karaoke bars, 3.0% brothels. -*Chlamydia*: 56.3% massage parlors, 40.6% karaoke bars, 69.9% brothels.
Zurita et al. (1997) [44]	Perù	51 female SWs	-HTLV-l: 13.7% -HTLV-2: 0.0%	-HIV-1: 0.0%	-
Broutetet et al. (1996) [45]	Brazil	496 female and 171 male SWs	-HTLV-1: 1.2 female, 0.6% male -HTLV-2: 0.2% female, 0.0% male	-HIV-1: 1.6% female, 2.9% male -HIV-2: 0.0%	-
Bellei et al. (1996) [46]	Brazil	653 female SWs	-HTLV-1: 2.8% -HTLV-2: 0.0%	-HIV-1: 3.6% -HIV-2: 0.0%	-HBV: 5.1% -HCV: 10.3%
Yoshida et al. (1987) [48]	Japan	237 female SWs	-HTLV-1: 5.9%	-HIV-1: 0.0%	-HBV: 34.2%

IM: immigrants; TP: Treponema Pallidum.

## Data Availability

Data sharing not applicable.
